# Supplementary figures and images for: Substrate stiffness regulates triple-negative breast cancer signaling through CXCR4 receptor dynamics
Source: Sci Rep. 2025 Aug 13;15:29621. doi: 10.1038/s41598-025-14495-x (PMC12343888; doi:10.1038/s41598-025-14495-x)

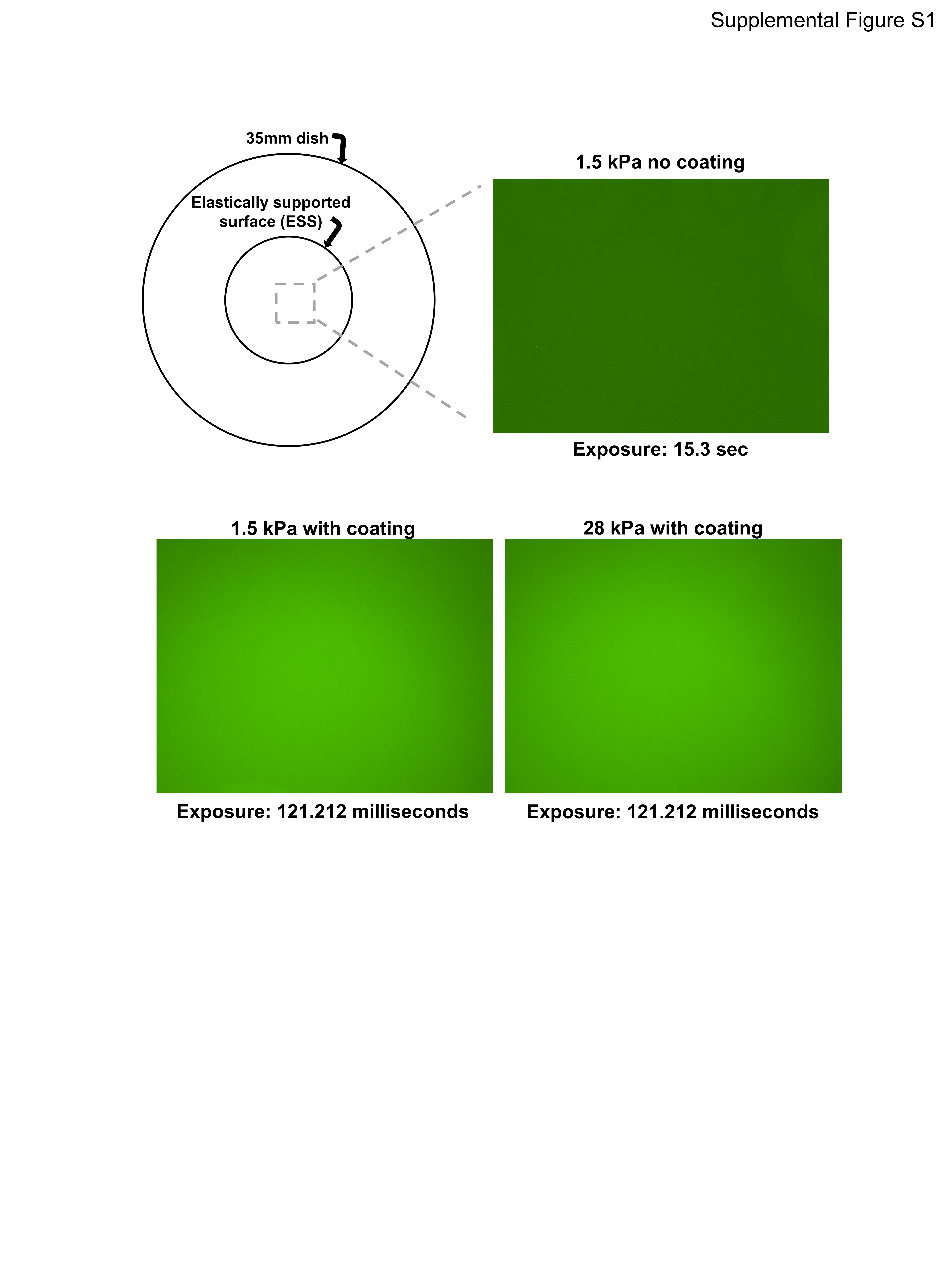

Supplement: Supplementary file 1 — Supplementary Material 1 [file 41598_2025_14495_MOESM1_ESM.tif]

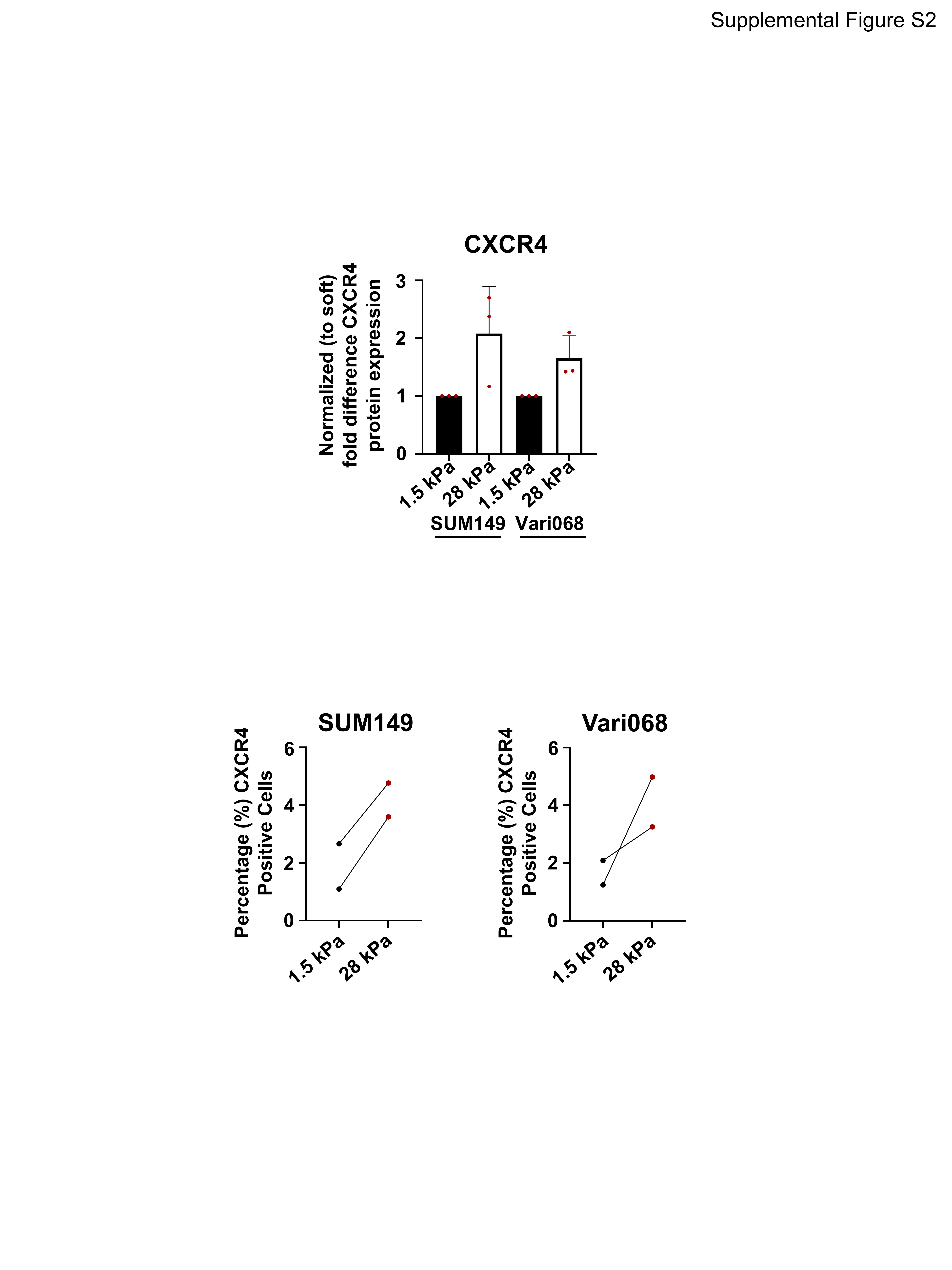

Supplement: Supplementary file 2 — Supplementary Material 2 [file 41598_2025_14495_MOESM2_ESM.tif]

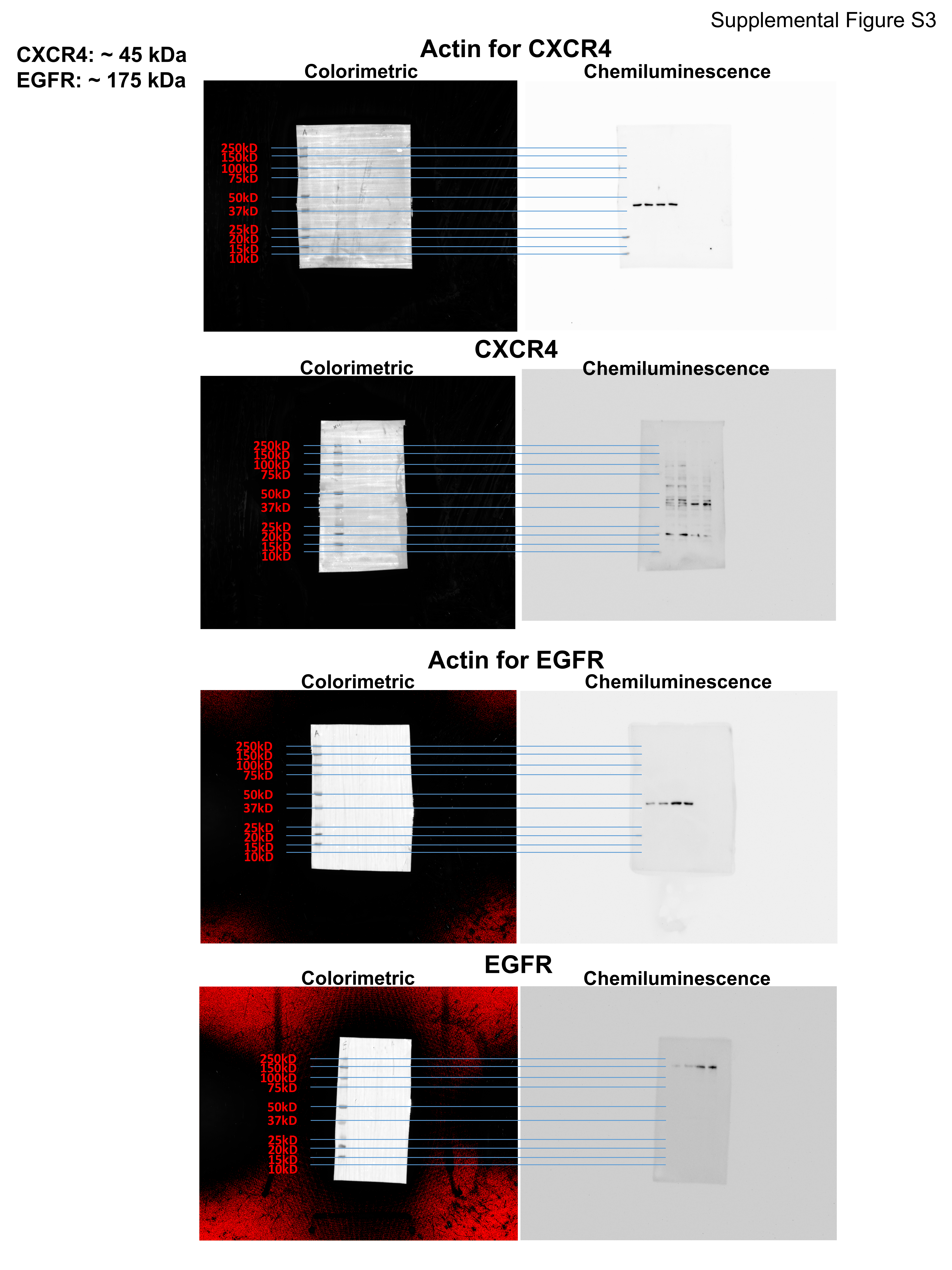

Supplement: Supplementary file 3 — Supplementary Material 3 [file 41598_2025_14495_MOESM3_ESM.tif]

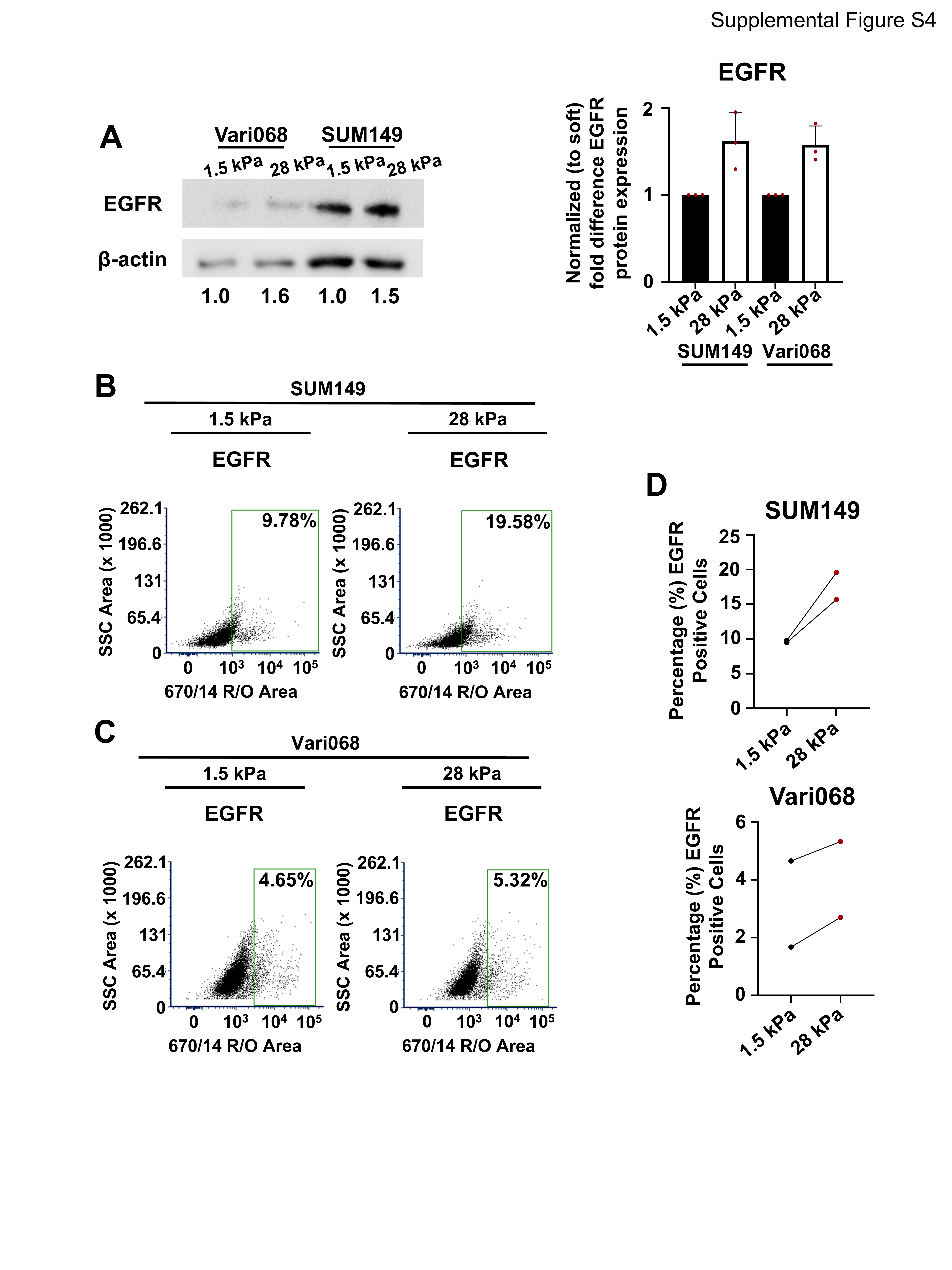

Supplement: Supplementary file 4 — Supplementary Material 4 [file 41598_2025_14495_MOESM4_ESM.tif]

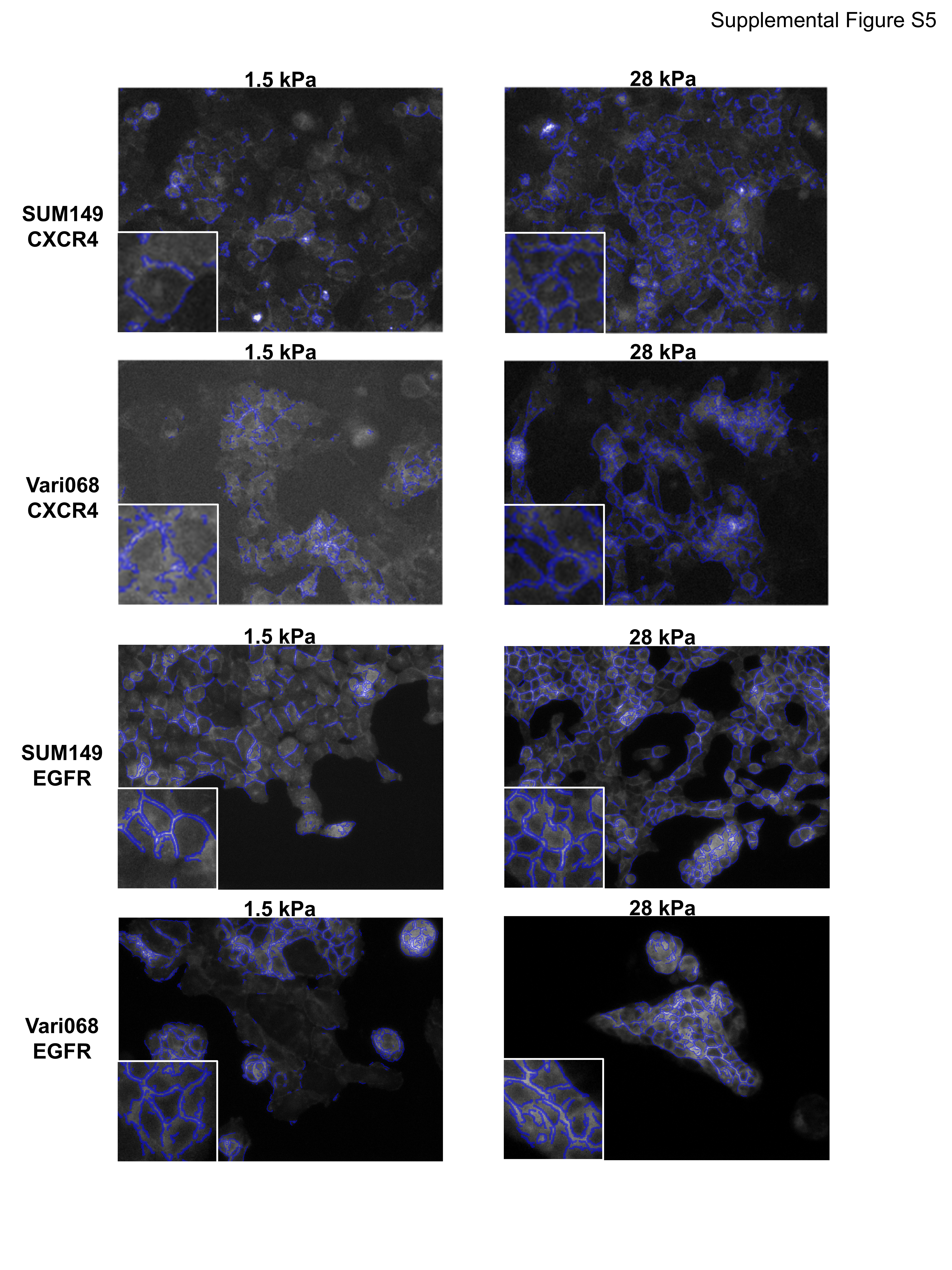

Supplement: Supplementary file 5 — Supplementary Material 5 [file 41598_2025_14495_MOESM5_ESM.tif]

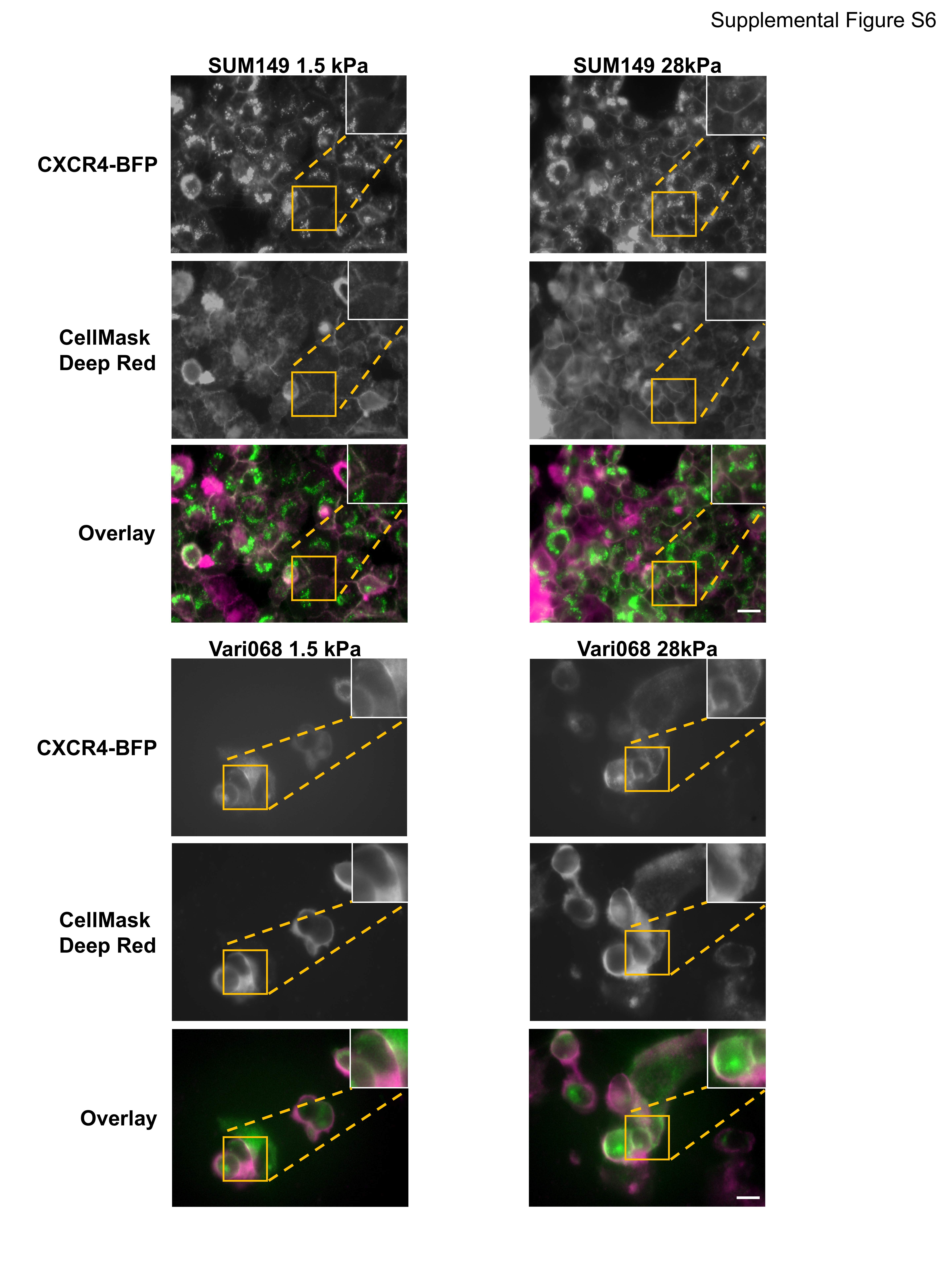

Supplement: Supplementary file 6 — Supplementary Material 6 [file 41598_2025_14495_MOESM6_ESM.tif]

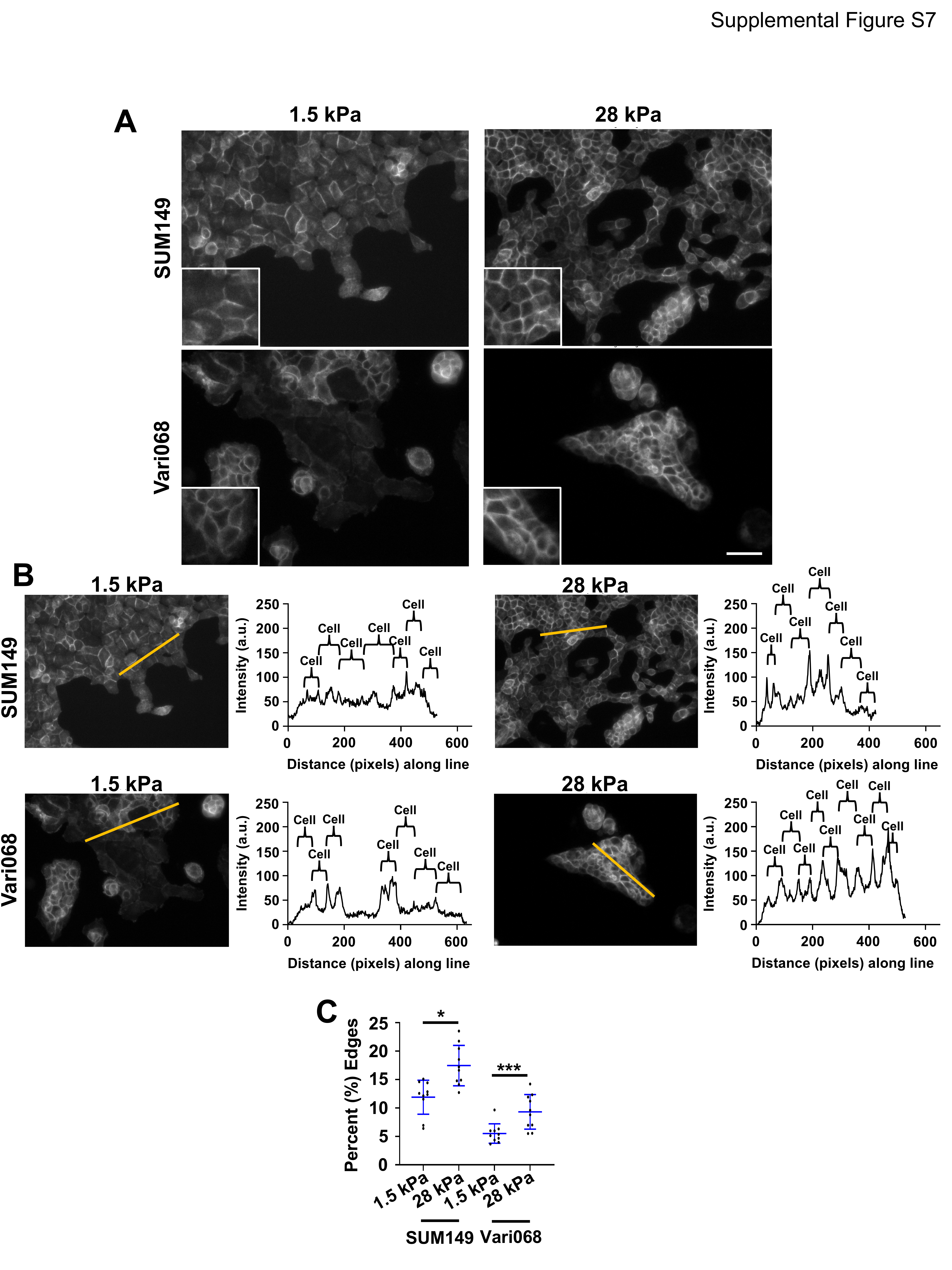

Supplement: Supplementary file 7 — Supplementary Material 7 [file 41598_2025_14495_MOESM7_ESM.tif]

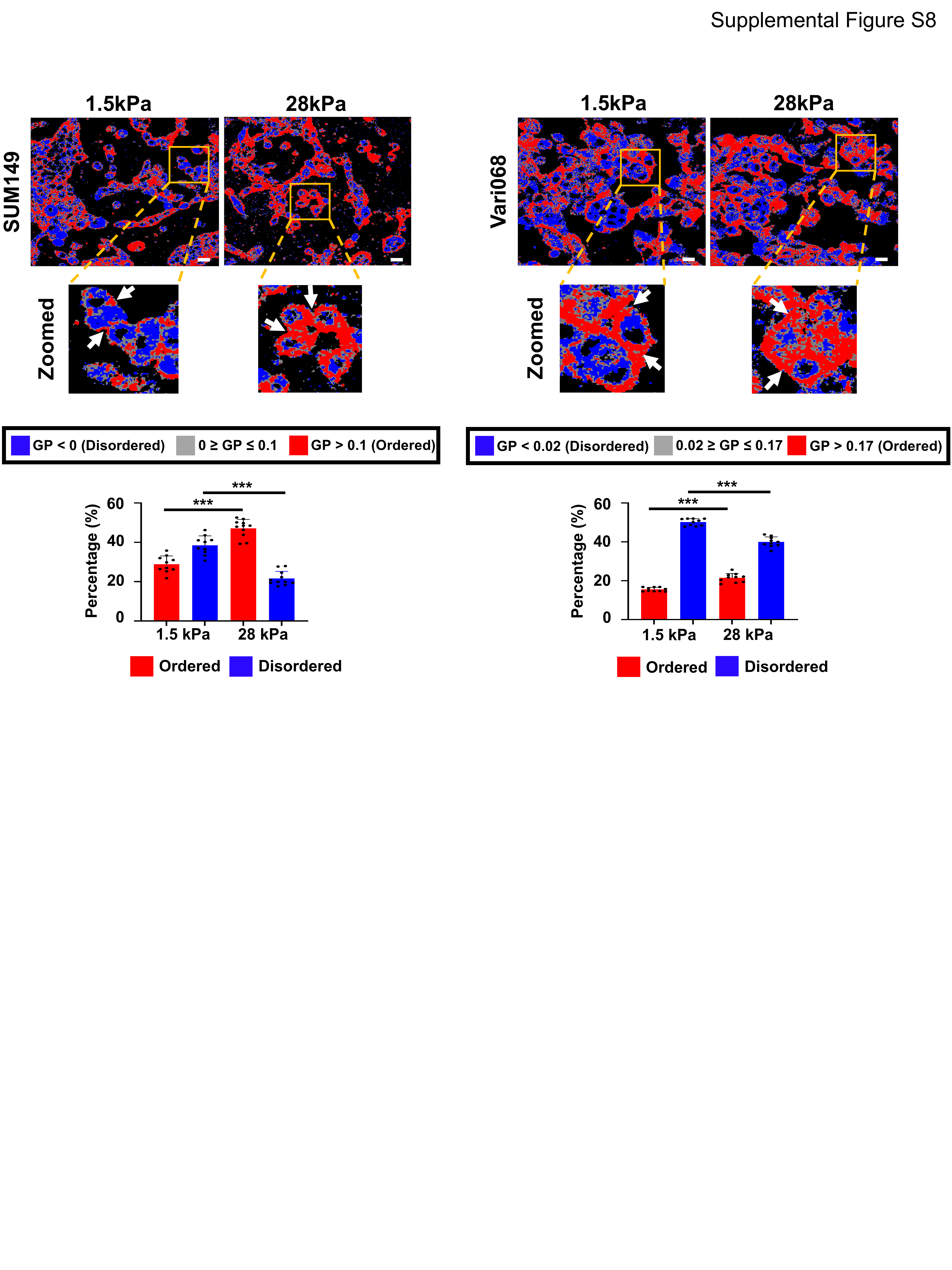

Supplement: Supplementary file 8 — Supplementary Material 8 [file 41598_2025_14495_MOESM8_ESM.tif]

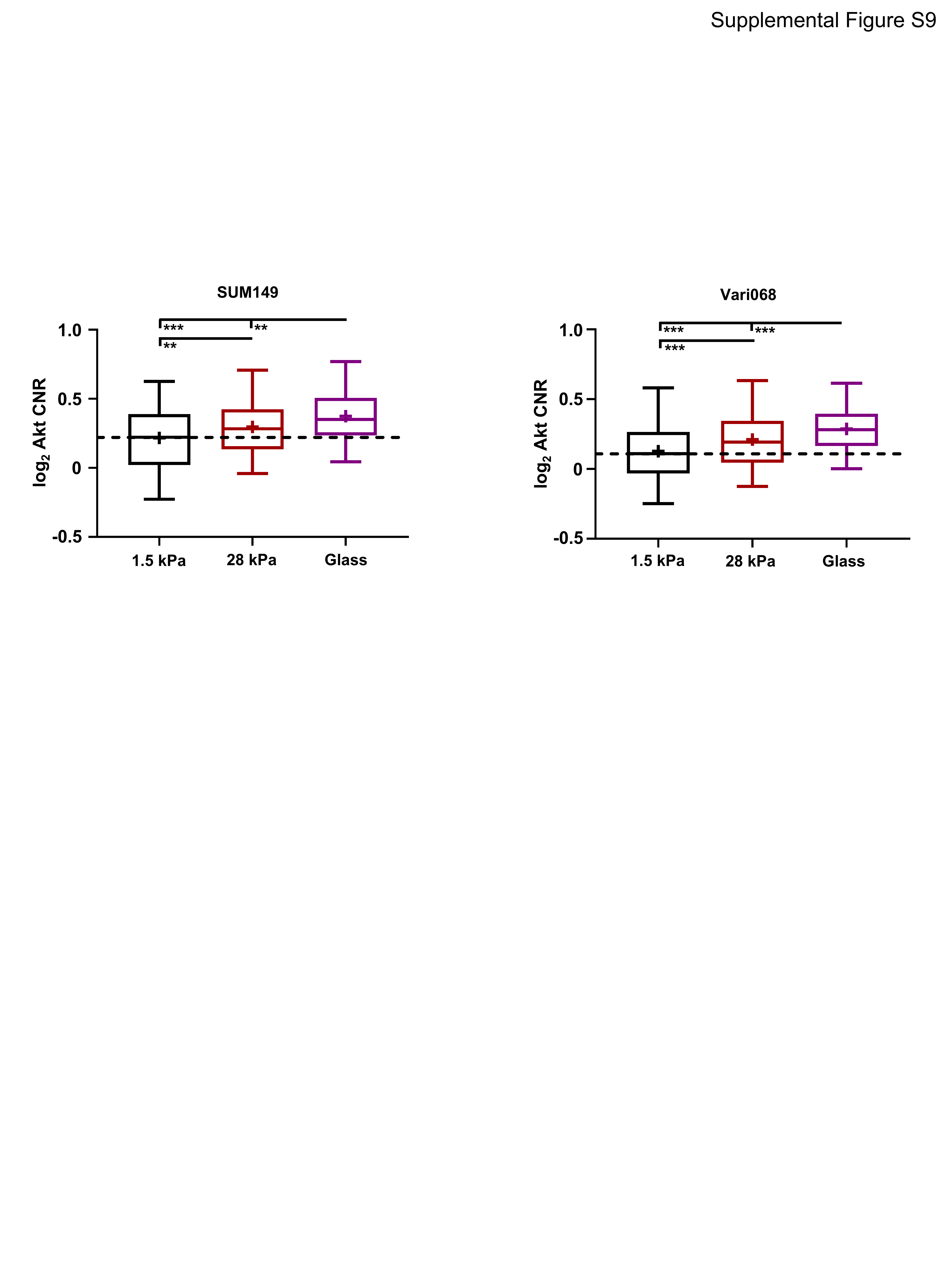

Supplement: Supplementary file 9 — Supplementary Material 9 [file 41598_2025_14495_MOESM9_ESM.tif]

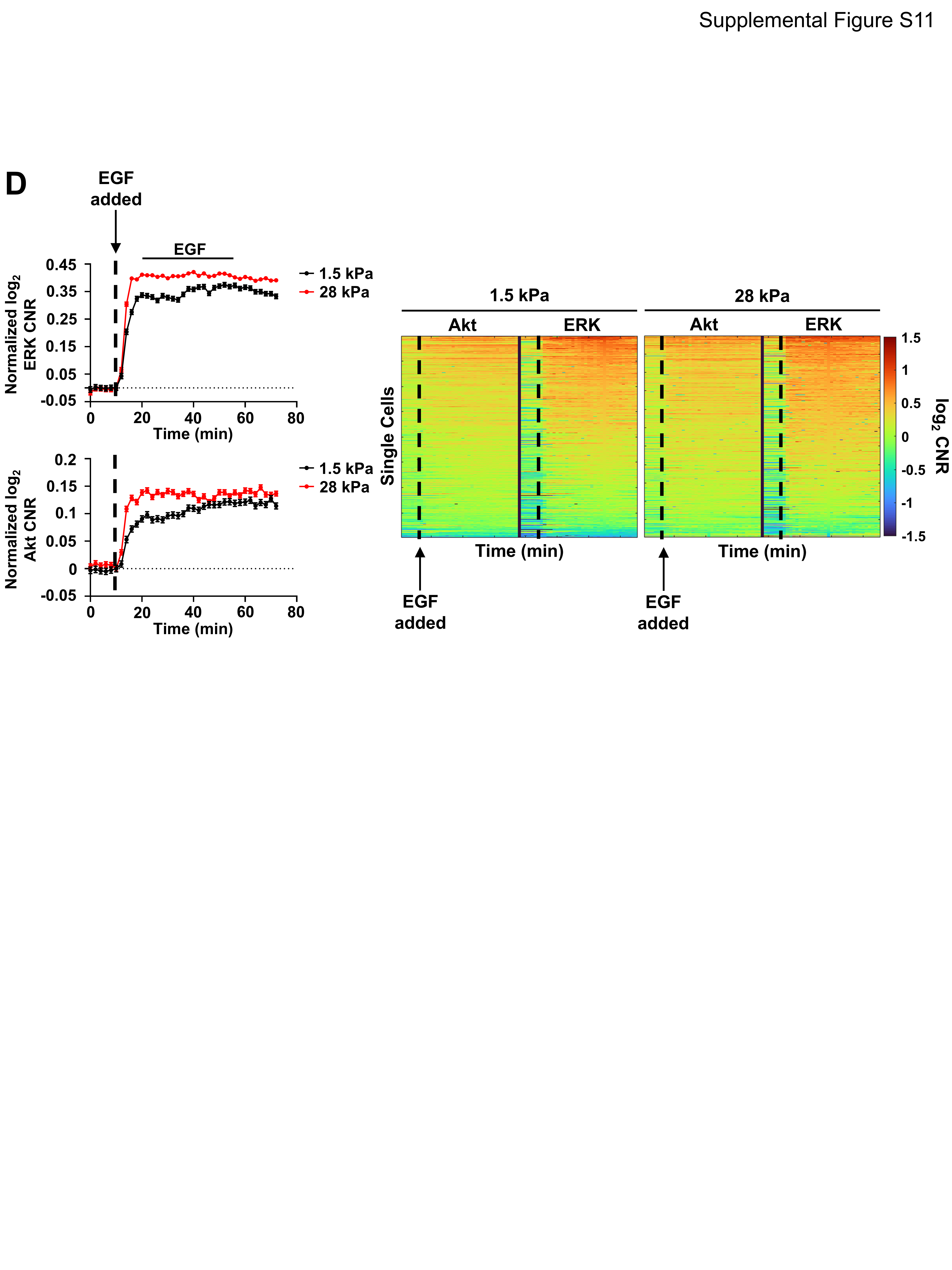

Supplement: Supplementary file 11 — Supplementary Material 11 [file 41598_2025_14495_MOESM11_ESM.tif]

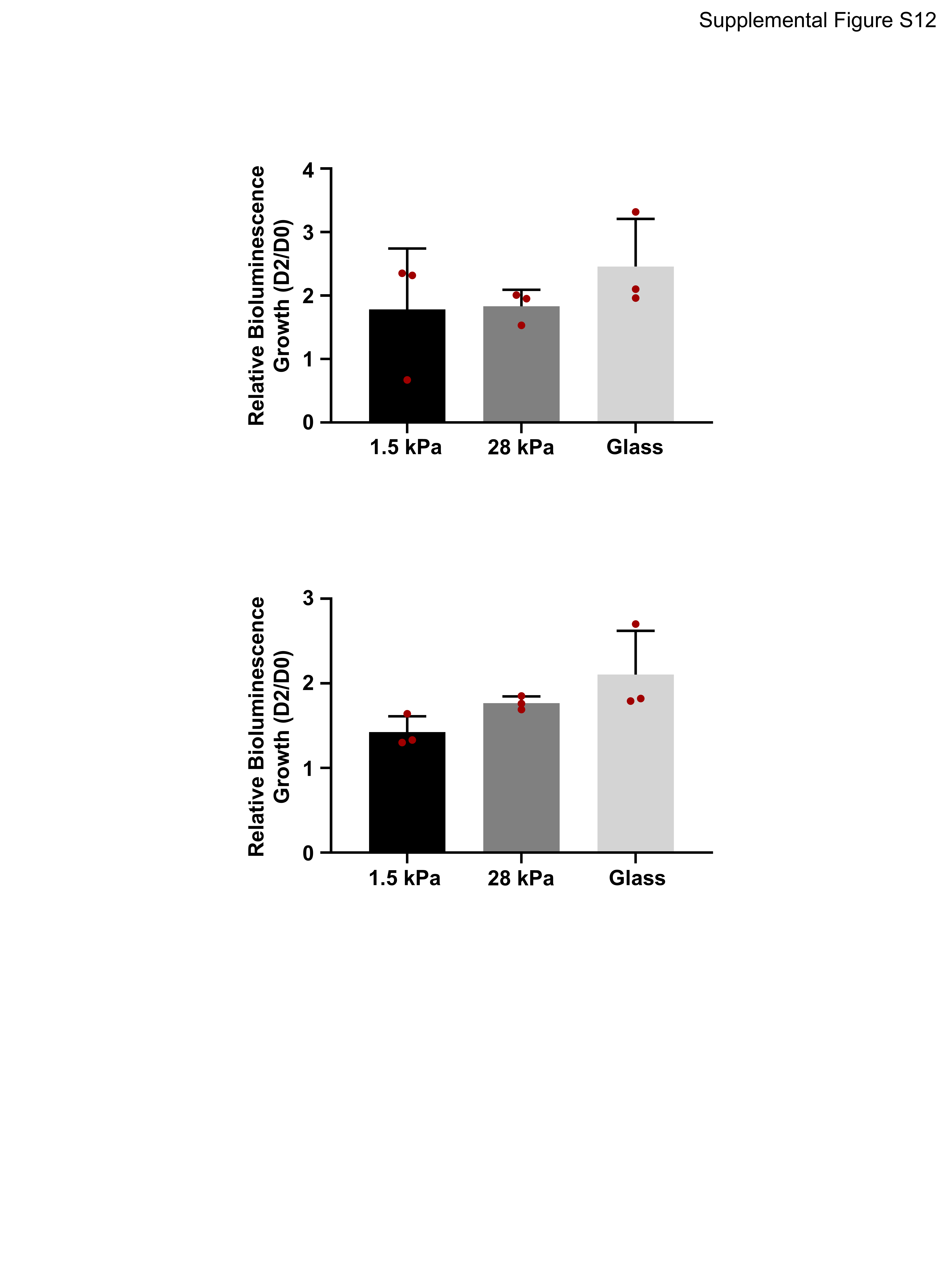

Supplement: Supplementary file 12 — Supplementary Material 12 [file 41598_2025_14495_MOESM12_ESM.tif]

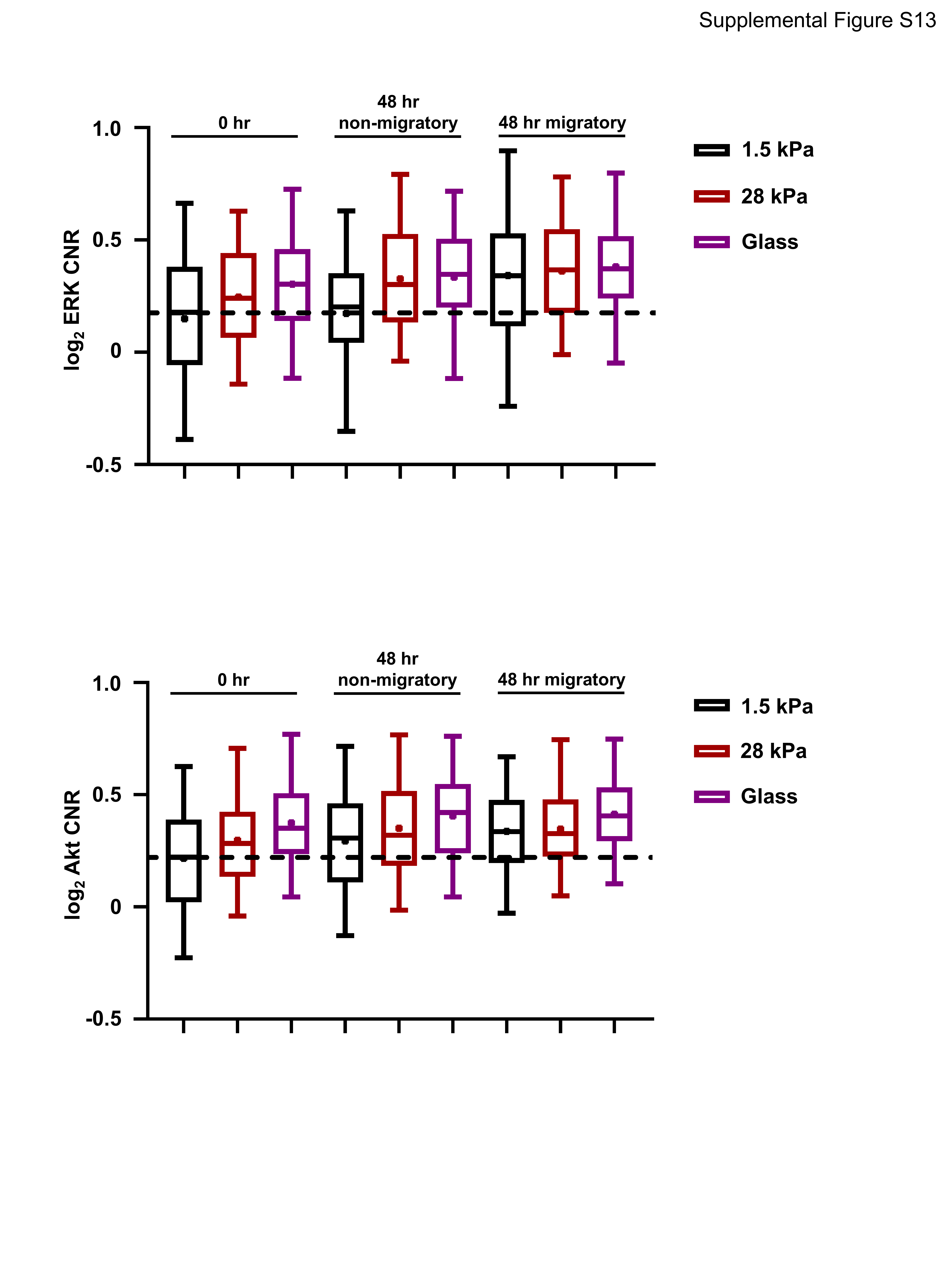

Supplement: Supplementary file 13 — Supplementary Material 13 [file 41598_2025_14495_MOESM13_ESM.tif]

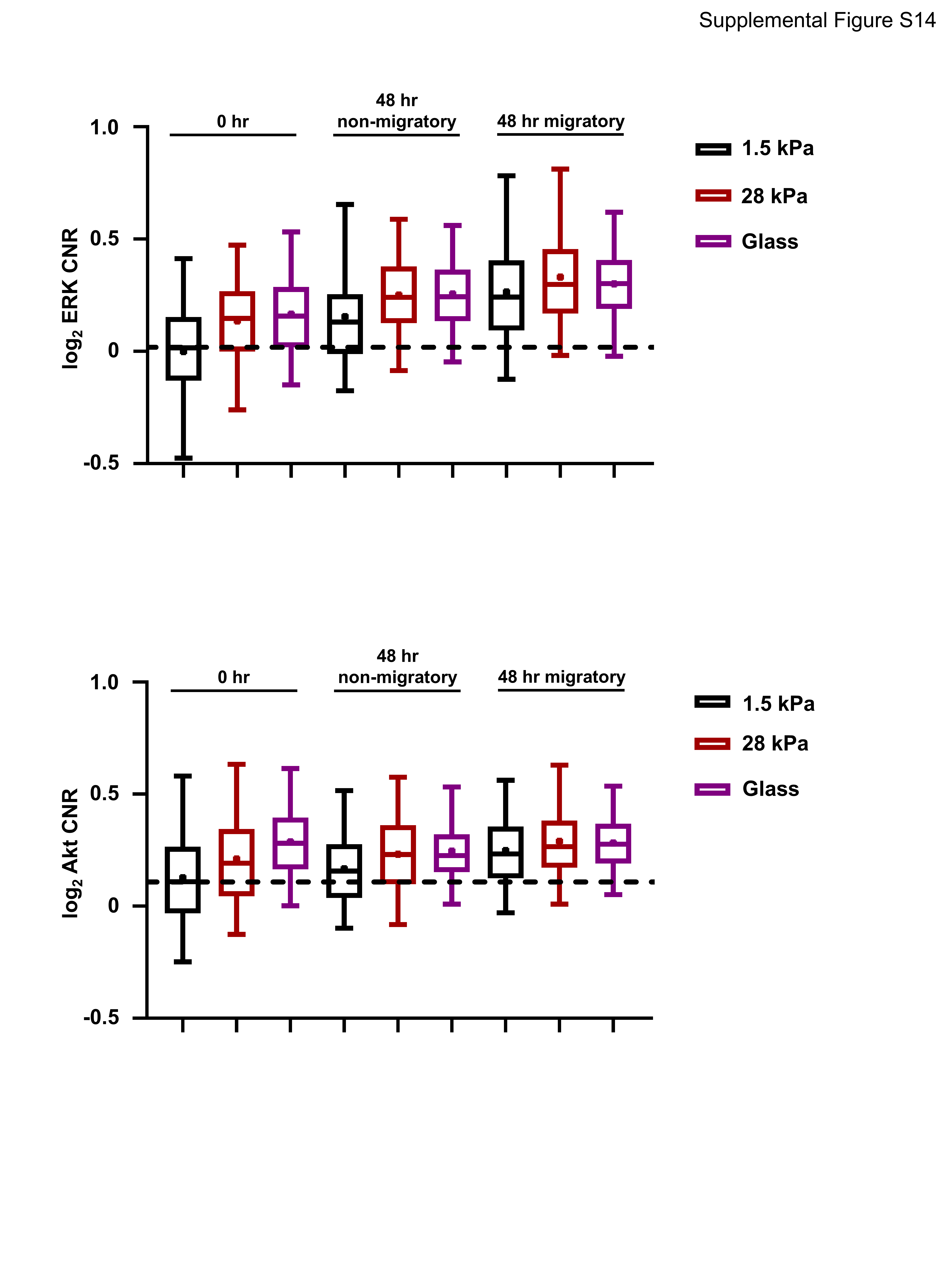

Supplement: Supplementary file 14 — Supplementary Material 14 [file 41598_2025_14495_MOESM14_ESM.tif]

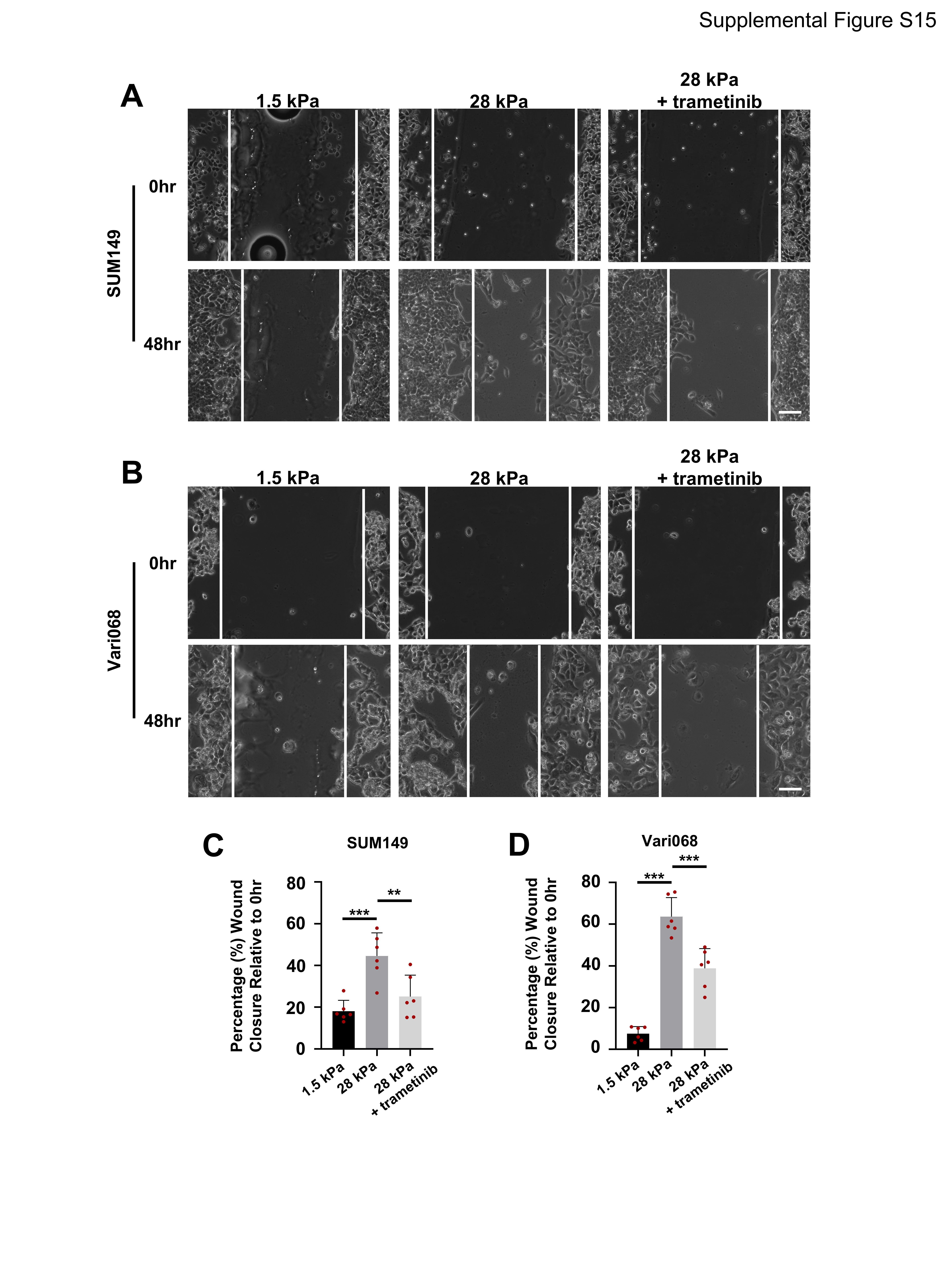

Supplement: Supplementary file 15 — Supplementary Material 15 [file 41598_2025_14495_MOESM15_ESM.tif]
