# Supplementary material for: Substrate stiffness regulates triple-negative breast cancer signaling through CXCR4 receptor dynamics
Source: Sci Rep. 2025 Aug 13;15:29621. doi: 10.1038/s41598-025-14495-x (PMC12343888; doi:10.1038/s41598-025-14495-x)
Supplement: Supplementary file 16 — Supplementary Material 16 [file 41598_2025_14495_MOESM16_ESM.docx]

**Supplemental Figure Legends**

**Supplemental Figure S1. Coating of ESS dishes with fluorescent fibrinogen.** ESS dishes were coated with fibrinogen conjugated to AlexaFluor 488 as described in the Materials and Methods section to confirm successful coating and imaged after washing.

**Supplemental Figure S2. Increased expression of CXCR4 in cells cultured on a stiffer matrix.** Top. Analysis of three independent replicates (*n* = 3) of CXCR4 levels in SUM149 and Vari068 cells cultured on a soft (1.5 kPa) or stiff (28 kPa) matrix normalized to β-actin for the same sample relative to cells cultured on a soft (1.5 kPa) matrix. Bottom. Graphs show matched total percentage of cells that express CXCR4 on soft or stiff substrates from each Flow Cytometry experiment from Figures 1E-F for SUM149 (**left**) and Vari068 (**right**).

**Supplemental Figure S3. Raw Western Blots for CXCR4 and EGFR.** Raw colorimetric (**left**) and chemiluminescence (**right**) images of Western Blots for CXCR4 (**top**) and EGFR (**bottom**).

**Supplemental Figure S4. Increased expression of EGFR in cells cultured on a stiffer matrix.** **A.** Representative Western blot image (**left**) and analysis of three independent replicates (*n* = 3, **right**) analysis of EGFR levels in SUM149 and Vari068 cells cultured on a soft (1.5 kPa) or stiff (28 kPa) matrix. Values under the blots show values of EGFR normalized to β-actin for the same sample relative to cells cultured on a soft (1.5 kPa) matrix. **B-C.** Flow cytometry plots for SUM149 (**B**) and Vari068 (**C**) cells show greater expression of EGFR in cells cultured on a stiff (28 kPa) versus soft (1.5 kPa) environment. (n ≥ 9,433 total cells analyzed in each condition). Cells stained with the IgG control antibody define the control gate. Similar results were obtained in one additional replicate. **D.** Graphs show matched total percentage of cells that express EGFR on soft or stiff substrates from each Flow Cytometry experiment from (**B**) and (**C**) for SUM149 (**top**) and Vari068 (**bottom**).

**Supplemental Figure S5. Cells cultured on a stiffer matrix have higher CXCR4 on cell edges.** Representative images of edge detection in SUM149 and Vari068 WT cells that stably express CXCR4-BFP for CXCR4 (**top**) and EGFR (**bottom**) cultured on 1.5 kPa (soft, **left**) and 28 kPa (stiff, **right**) matrices under baseline signaling conditions described in materials and methods. We analyzed cell edges with custom MATLAB code.

**Supplemental Figure S6. CXCR4 localizes to the cell membrane.** Representative images of SUM149 (**top**) and Vari068 (**bottom**) cells stably expressing CXCR4 (green) and stained with CellMask plasma membrane marker (magenta) cultured on 1.5 kPa (soft, **left**) and 28 kPa (stiff, **right**) matrices under baseline signaling conditions described in materials and methods. Overlay images are pseudocolored as green and magenta to facilitate viewing the colocalization. Scale bar is 20 µm.

**Supplemental Figure S7. Cells cultured on a stiffer matrix have higher EGFR on cell edges.** **A.** Representative images of SUM149 and Vari068 WT cells stained with EGFR-AlexaFluor 647 cultured on 1.5 kPa (soft, **left**) and 28 kPa (stiff, **right**) matrices under baseline signaling conditions described in materials and methods. Scale bar is 50 µm. **B.** Representative line intensity and correlated histograms showing increased edge intensity of EGFR in cells cultured on a 28 kPa matrix. Images in A and from B come from the same experiment. C. Graphs show quantification of the edges of EGFR in cells cultured on 1.5 kPa (soft) versus 28 kPa (stiff) matrices. *: p < 0.05, ***: p < 0.001.

**Supplemental Figure S8. Increased substrate stiffness increases membrane order.** Representative images of SUM149 or Vari068 cells stained with laurdan. Images show pseudocolored general polarization (GP) values. Red color indicates areas of high membrane order and less dynamics, whereas blue color denotes areas of low membrane order and more dynamics. Gray color indicates areas between the ordered and disordered phases based upon an arbitrary cutoff used for all groups. Arrows on zoomed in image point to cell-cell junctions. Scale bar is 20 μm. Graphs show mean + SD for the percentage of all ordered and disordered pixels for each image above (n = 10 images per group). Similar results were obtained in two additional replicates. ***: *p* < 0.001.

**Supplemental Figure S9. Enhanced substrate stiffness increased baseline Akt signaling in TNBC cells.** Box plot and whiskers for quantified log_2_ cytoplasmic/nuclear fluorescence intensities (cytoplasmic-to-nuclear ratio, CNR) for Akt activity in SUM149 (**left**) and Vari068 (**right**) at the time of making the wound in a monolayer of confluent cells (0 hr) (*n* ≥ 180 cells per group, three combined independent replicates). Line within the box denotes the median, and the “+” symbol denotes the mean. Dashed line represents the median of cells cultured on soft (1.5 kPa) ESS dishes. **p* < 0.05, ***p* < 0.01, ****p* < 0.0001.

**Supplemental Figure S10. Enhanced matrix stiffness promotes single cell ERK signaling in response to serum.** We quantified activation of Akt and ERK in single SUM149 cells by imaging KTRs. Graphs show mean ± SEM for activation of Akt and ERK in an average cell in each condition in response to serum (10%) expressed as log_2_ of cytoplasmic to nuclear ratio (CNR) of fluorescence intensities normalized to the KTR value of the image before stimulus (t = 6) (*n* ≥ 2,283 cells per group). Dashed vertical line denotes the time for adding a stimulus. Right. Single cell time tracks show activation of Akt and ERK in SUM149 cells quantified as the change in log_2_ CNR for each KTR in individual cells and displayed on a pseudocolor scale. A red color signifies increased Akt or ERK activity, while a blue color signifies decreased Akt or ERK activity. Compared to a soft environment, SUM149 cells cultured on a stiffer environment showed greater ERK signaling in response to serum. Dashed vertical line denotes the time point where the stimulus was added. Similar results were obtained in two additional replicates.

**Supplemental Figure S11. Enhanced matrix stiffness promotes single cell ERK signaling in response to EGF.** We quantified activation of Akt and ERK in single SUM149 cells by imaging KTRs. Graphs show mean ± SEM for activation of Akt and ERK in an average cell in each condition in response to EGF (50 ng/mL) expressed as log_2_ of cytoplasmic to nuclear ratio (CNR) of fluorescence intensities normalized to the KTR value of the image before stimulus (t = 6) (*n* ≥ 2,283 cells per group). Dashed vertical line denotes the time for adding a stimulus. Right. Single cell time tracks show activation of Akt and ERK in SUM149 cells quantified as the change in log_2_ CNR for each KTR in individual cells and displayed on a pseudocolor scale. A red color signifies increased Akt or ERK activity, while a blue color signifies decreased Akt or ERK activity. Compared to a soft environment, SUM149 cells cultured on a stiffer environment showed greater ERK signaling in response to serum. Dashed vertical line denotes the time point where the stimulus was added. Similar results were obtained in two additional replicates.

**Supplemental Figure S12. Substrate stiffness does not significantly change growth.** Relative growth of SUM149 (**top**) and Vari068 (**bottom**) cells on ESS dishes of 1.5 kPa or 28 kPa, or on glass. Graphs show mean values + SD for bioluminescence signal after 48 hrs (Day 2) relative to initial bioluminescence signal after seeding (Day 0) (n = 3). Similar results were obtained in two additional replicates.

**Supplemental Figure S13. Increased matrix stiffness drives Akt and ERK signaling in SUM149 cells.** Box plot and whiskers for quantified log_2_ cytoplasmic/nuclear fluorescence intensities (cytoplasmic-to-nuclear ratio, CNR) for ERK (**top**) and Akt (**bottom**) activities based on the imaging from Fig 5E. Line within the box denotes the median, and the “+” symbol denotes the mean. Dashed line represents the median of the control group at the initial time point. See Supplemental Tables 1 and 2 for statistical comparisons.

**Supplemental Figure S14. Increased matrix stiffness drives Akt and ERK signaling in Vari068 cells.** Box plot and whiskers for quantified log_2_ cytoplasmic/nuclear fluorescence intensities (cytoplasmic-to-nuclear ratio, CNR) for ERK (**top**) and Akt (**bottom**) activities based on the imaging from Fig 5F. Line within the box denotes the median, and the “+” symbol denotes the mean. Dashed line represents the median of the control group at the initial time point. See Supplemental Tables 3 and 4 for statistical comparisons.

**Supplemental Figure S15. ERK signaling inhibition reduces migration on a stiff substrate.** **A-B**. Representative wound healing images of SUM149 (**A**) and Vari068 (**B**) cells on ESS dishes with different stiffnesses at the time the wound was made (0 hr) and after 48 hr. Scale bar is 100 µm. **C-D.** Percentages of wound closure for SUM149 (**C**) and Vari068 (**D**) are presented as mean values ± SD (*n* = 6, two combined independent replicates). **: p < 0.01, ***: p < 0.001.
