# Supplementary material for: Substrate stiffness regulates triple-negative breast cancer signaling through CXCR4 receptor dynamics
Source: Sci Rep. 2025 Aug 13;15:29621. doi: 10.1038/s41598-025-14495-x (PMC12343888; doi:10.1038/s41598-025-14495-x)
Supplement: Supplementary file 17 — Supplementary Material 17 [file 41598_2025_14495_MOESM17_ESM.docx]

| **Supplemental Table S1. Features of the three datasets used for bioinformatics analysis to determine drivers of enhanced Akt and ERK signaling on a stiff environment.** | | | | |
| --- | --- | --- | --- | --- |
| **Dataset** | GSE107063 | | | GSE93529 |
| **Platform** | Affymetrix array | | | Affymetrix array |
| **Cell Line Used** | MDA-MB-453 | | | MDA-MB-231 |
| **Conditions** | Fibronectin coated polyacrylamide gels; 5 and 30 kPa and glass | | | 0.7 kPa Hydrogels vs. TCPS |
| **Culture length** | 5 days | | | 2 days |
| **Replicates** | N = 4/condition | | | N = 4/condition |
| **Comparisons Used** | Glass v. Soft | Glass v. Mid | Mid v. Soft | Plastic v. Soft |
| **Genes UP** | 283 | 38 | 36 | 20 |
| **Genes DOWN** | 86 | 6 | 25 | 36 |
