# Supplementary material for: Substrate stiffness regulates triple-negative breast cancer signaling through CXCR4 receptor dynamics
Source: Sci Rep. 2025 Aug 13;15:29621. doi: 10.1038/s41598-025-14495-x (PMC12343888; doi:10.1038/s41598-025-14495-x)
Supplement: Supplementary file 21 — Supplementary Material 21 [file 41598_2025_14495_MOESM21_ESM.docx]

| **Supplemental Table S5. Calculated *p*-values for the Akt KTR in Vari068 cells from data in Supplemental Fig S10 using Tukey’s multiple comparisons test.** | | | | | | | | | | |
| --- | --- | --- | --- | --- | --- | --- | --- | --- | --- | --- |
|  |  | **1.5 kPa** | | | **28 kPa** | | | **Glass** | | |
|  |  | **0 hr** | **48 hr**  **non-mig** | **48 hr mig** | **0 hr** | **48 hr**  **non-mig** | **48 hr mig** | **0 hr** | **48 hr**  **non-mig** | **48 hr mig** |
| **1.5 kPa** | **0 hr** |  |  | ******* | ******* | ******* | ******* | ******* | ******* | ******* |
|  | **48 hr non-mig** |  |  | ******* |  | ***** | ******* | ******* | ******* | ******* |
|  | **48 hr mig** |  |  |  |  |  |  |  |  |  |
| **28 kPa** | **0 hr** |  |  |  |  |  | ******* | ******* |  | ****** |
|  | **48 hr non-mig** |  |  |  |  |  |  |  |  |  |
|  | **48 hr mig** |  |  |  |  |  |  |  |  |  |
| **Glass** | **0 hr** |  |  |  |  |  |  |  |  |  |
|  | **48 hr non-mig** |  |  |  |  |  |  |  |  |  |
|  | **48 hr mig** |  |  |  |  |  |  |  |  |  |
| **Non-mig = non-migratory cell, mig = migratory cell, * = *p* < 0.05, ** = *p* < 0.01, *** = *p* < 0.001.** | | | | | | | | | | |
